# Affinity-Based Copolymer Coating for Oriented Protein Immobilization in Biosensor Development

**DOI:** 10.3390/bios15100670

**Published:** 2025-10-04

**Authors:** Lorenzo Zarini, Thomas Carzaniga, Morena Pirotta, Francesco Damin, Dario Brambilla, Marcella Chiari, Ivan Bassanini, Paola Gagni, Alessandro Mussida, Luca Casiraghi, Marco Buscaglia, Laura Sola

**Affiliations:** 1 Institute of Chemical and Technological Sciences “Giulio Natta”, National Research Council of Italy, Via Mario Bianco 9, 20131 Milan, Italy; lorenzo.zarini@scitec.cnr.it (L.Z.); morena.pirotta@scitec.cnr.it (M.P.); francesco.damin@scitec.cnr.it (F.D.); dario.brambilla@scitec.cnr.it (D.B.); marcella.chiari@scitec.cnr.it (M.C.); ivan.bassanini@scitec.cnr.it (I.B.); paola.gagni@scitec.cnr.it (P.G.); alessandro.mussida@gmail.com (A.M.); 2 Department of Molecular and Translational Medicine, University of Brescia, 25121 Brescia, Italy; 3 Department of Medical Biotechnology and Translational Medicine, Università degli Studi di Milano, 20054 Segrate, Italy; thomas.carzaniga@unimi.it (T.C.); luca.casiraghi@unimi.it (L.C.); marco.buscaglia@unimi.it (M.B.)

**Keywords:** surface coating, protein immobilization, NTA, his-tag protein, microarray, biosensor

## Abstract

Effective protein immobilization is a critical step in biosensor development, as it ensures the stability, functionality, and orientation of biomolecules on the sensor surface. Here, we present a novel affinity-based terpolymer coating designed to enhance protein immobilization for biosensor applications. The novelty lies in the incorporation of nitrilotriacetic acid (NTA) ligands directly into the polymeric chains, facilitating histidine-tagged protein oriented binding through a robust metal-chelating interaction. To validate the system, magnetic microbeads coated with the polymer were tested for their ability to bind native and His-tagged proteins. The results demonstrated the superior binding capacity, enhanced stability, and reversibility of the interactions compared to traditional coatings, which immobilize proteins through nucleophile reactions with amine residues. Moreover, enzyme immobilization tests confirmed that the polymer preserves enzymatic activity, highlighting its potential for biosensor applications requiring functional biomolecules. This innovative polymeric coating offers a fast, versatile, and scalable solution for next-generation biosensor platforms, paving the way for improved sensitivity, reliability, and accessibility in diagnostic and analytical technologies.

## 1. Introduction

The immobilization of biomolecules, especially proteins, represents an essential step in the development of biosensors. Indeed, to ensure the high sensitivity, specificity, and reliability of the biosensor, an effective immobilization strategy must not only secure the stable binding of the probe to the surface, but also preserve its three-dimensional structure and, ideally, promote the correct orientation. This arrangement facilitates an optimal interaction with its counterpart, enhancing the biosensor’s performance. The literature reports several methods to pursue this goal, which are usually based on covalent binding. In fact, the physical adsorption of biomolecules on the sensors’ surface can easily lead to desorption and non-specific immobilization, drastically reducing the assay’s performance. On the contrary, covalent strategies, which exploit reactions between the functional groups of the proteins (e.g., amino, carboxyl, or thiol groups) and reactive groups on the surface (e.g., carboxyl, hydroxyl, or epoxy groups), assure binding stability, although a proper orientation is not always achieved. Among several strategies, click chemistry has been widely adopted to correctly orient the probe onto the surface. However, this method usually requires a modification of the biomolecule to insert click functionalities in order for it to react with the corresponding groups on the surface of the biosensor. Since the click moieties are not naturally present in proteins, this strategy assures a proper orientation and drastically reduces non-specific binding; however, it can be an easy source of 3D-structure loss, negatively affecting the efficiency of the biorecognition interaction. A good compromise lies in the affinity-based immobilization techniques which leverage specific and reversible interactions between a probe (e.g., enzyme, antibody, or DNA) and a surface-bound ligand [1]. Among these, the most widely recognized and utilized strategy is the streptavidin–biotin interaction, known for being one of the strongest non-covalent bonds. Similarly, other mechanisms employed in biosensor development include the interaction of Protein A or Protein G with the Fc region of antibodies, ensuring specific and oriented binding, and the interaction of lectins with carbohydrates, which enables selective binding to glycoproteins or glycosylated compounds. Another prominent strategy, based on the principles of immobilized metal ion affinity chromatography (IMAC), involves the interaction between transition metal cations (e.g., Cu^2+^, Ni^2+^, Zn^2+^, and Co^2+^) and accessible metal-binding amino acid residues such as histidine. In this strategy, the binding between the metal ion and the imidazole moieties of the histidine tag (usually a short sequence of six histidines incorporated into proteins through a genetic engineering process) is mediated by the presence of a metal chelating ligand fixed to the biosensor’s surface. The most-used ligands are iminodiacetic acid (IDA), tetradentate nitrilotriacetic acid (NTA), and pentadentatetris(carboxymethyl)ethylene diamine, even if alternative ligands have been introduced, such as the macrocycle triazacyclononane described by Johnson and collaborators [2]. This technology is a powerful tool for the single-step isolation and purification of proteins and enzymes modified at the N- or C-terminus with a series of histidine residues [3]. Furthermore, thanks to the high binding affinity between NTA ligands and histidine, this strategy has found a wide application also in the development of sensors based on Surface Plasmon Resonance (SPR) electrochemistry and fluorescence detection [2,3,4].

The binding of the ligand to the sensors’ surface through the NTA strategy has mostly been obtained by the formation of a Self-Assembled Monolayer (SAM) [5,6,7,8], which is known to present several drawbacks compared to 3D chemistries, due to the reduced stability of the immobilized molecule and the limited density and restricted surface area for probe attachment. On the other hand, the literature reports examples of 3D architecture. For example, after the introduction of the dextran matrix by Biacore, techniques such as Surface Plasmon Resonance (SPR) have exploited modifications of a hydrogel network with NTA ligands to immobilize proteins on the surface of their chips. Similarly, Gautrot and collaborators reported on the functionalization of poly(oligoethylene glycol methacrylate) poly(hydroxyethyl methacrylate) polymer brushes with NTA groups [9]. Oshige et al. have introduced NTA-modified chitosan surfaces, whose production relies on surface silanization, an overnight treatment with glutaraldehyde, a second overnight incubation in chitosan, and finally functionalization with NTA [10]. More recently, Kaufmann and colleagues reported on the synthesis of microgels with NTA moieties to immobilize proteins to perform cell-free protein synthesis [11]. Castro-Hinojosa and collaborators presented a strategy to modify magnetic nanoparticles using a pre-formed NTA-M^2+^ complex and polyethylene glycol (PEG) molecules for the oriented immobilization of cadherins which strictly require orientation to maintain their activity [12]. Although promising, the production of such surfaces is quite complicated and time consuming.

Our lab has dedicated great efforts to the development of surface chemistry for proper protein immobilization, introducing a family of *N*, *N*-dimethylacrylamide (DMA) copolymers able to form a nanometric functional hydrophilic film on different substrates. This class of synthetic copolymers, namely MCP polymers, is constituted by DMA, *N*-acryloylsuccinimide (NAS), and 3-(Trimethoxysylil propyl) methacrylate (MAPS) in different molar fractions. Each monomer in the polymer imparts unique properties: the amphiphilic DMA backbone plays a key role in substrate adsorption via weak interactions such as hydrogen bonding and Van der Waals forces. The NAS groups, present in a minor molar fraction (2–10%), facilitate nucleophilic substitution reactions with amino-bearing molecules, enabling the covalent attachment of biological probes or post-polymerization modifications. Lastly, the MAPS monomer, containing silane groups, ensures the polymer anchors securely to silicon-based surfaces through covalent bond formation. This copolymer assures the formation, in a few minutes, of a hydrophilic nanometric film onto the surface of different materials (glass, silicon, thermoplastics, PDMS, gold) with different geometries (planar or spherical, such as magnetic beads) through the combination of physic-chemisorption [13].

Here we present a terpolymer which exploits the NTA–histidine interaction to bind proteins onto biosensors’ surfaces. The novelty lies in the incorporation of the metal chelating agent (NTA) directly into the polymeric chains. In particular, the NTA ligand, modified with an azido moiety, is inserted through a click chemistry reaction with the DBCO groups pending from the *N*, *N*-dimethylacrylamide polymer chain. Magnetic microbeads were coated and functionalized with the new coating to assess the ability to bind both native and His-tagged proteins compared to the original polymer that lacked the NTA ligand. Comprehensive testing was conducted to evaluate the coated surface’s protein-loading capacity and the reversibility of the binding interaction. The binding of proteins onto the newly introduced surface was analyzed using the Reflective Phantom Interface (RPI) technique, which analyzes the light reflected by a flat surface of glass with an anti-reflective layer spotted with specific probes [14]. This analysis confirmed an enhanced binding efficiency when His-tagged proteins were used as probes. To underscore the importance of effective immobilization, an enzyme was immobilized as a probe, and its activity was successfully retained, demonstrating the functionality of the coating in preserving enzymatic activity.

## 2. Materials and Methods

All reagents were of the highest purity grade from commercial suppliers. (*N-N*-bis[carbossimetil]) L-lysine, copper sulfate, potassium carbonate, nickel sulfate, 1H-imidazole-1-sulfonyl azide, α, α′-Azoisobutyronitrile *N*,*N*-dimethylacrylamide, 3-(Trimethoxysilyl)propyl methacrylate), glycidyl methacrylate, and dibenzocyclooctyne-amine were purchased from Sigma-Aldrich (St Louis, MO, USA). *N*-acryloyloxysuccinimide was synthesized according to the procedure reported in [15]. Alt-R A.s. Cas12a (Cpf1) Ultra, Alt-R A.s. Cas12a crRNA (sgRNA1), DNaseAlert substrate™, DNaseAlert Kit (Nuclease) were all provided by Integrated DNA Technologies, Inc. (IDT, Coralville, IA, USA). Synthetic DNA was provided by Metabion International AG (Planegg, Germany) with the following sequences: 1: Cy3-5_′-TTTTT-3_′-BHQ2 (report sequence); 5′-GCC TTC GGG TTG TAA AGT ACT TTC AGC GGG GAT GAA GGG AGT AAA GTT AAT ACC TTT GCT CAT TGA CGT TAC CCG CAG AAG AAG CAC CGG CTA ACT CCG TGC-3′ 5′-GCA CGG AGT TAG CCG GTG CTT CTT CTG CGG GTA ACG TCA ATG AGC AAA GGT ATT AAC TTT ACT CCC TTC ATC CCC GCT GAA AGT ACT TTA CAA CCC GAA GGC-3′ (dsDNA Target); AlTR1/rUrArArUrUrUrCrUrArCrUrCrUrUrGrUrArGrArUrArGrCrGrGrGrGrArUrGrArArGrGrGrArGrUrArA/AlTR2 (sgRNA1). Fluorescence measurements in vial were recorded with the Denovix QFX fluorometer (Wilmington, DE, USA). Spotting was performed using SciFLEXARRAYER S12 (Scienion, Berlin, Germany). NMR spectra were recorded with a Bruker AC spectrometer (400 MHz) in CDCl_3_ and D_2_O. Dynabeads^®^ MyOne™ SILANE and Pierce™ BCA Protein Assay Kit were purchased from Thermo Fisher Scientifics (Waltham, MA, USA).

More details on the synthesis, protocols, and experimental setup are reported in the Supplementary Materials (SM).

### 2.1. Synthesis of (S)-2,2′-((5-Azido-1-carboxypentyl)azanediyl)diacetic Acid

In a 50 mL round-bottom flask, (*N-N*-bis[carboxymethyl]) L-lysine (0.581 g, 2.21 mmol) was dissolved in water (17.5 mL, 130 mM). CuSO_4_ (0.012 g, 0.044 mmol) and K_2_CO_3_ (1.38 g, 9.97 mmol) were added and stirred until dissolved. 1H-imidazole-1-sulfonyl azide (0.557 g, 2.66 mmol) and acetonitrile (5 mL) were then added, and the mixture was stirred overnight at room temperature. After solvent removal, the crude was purified via aqueous workup, acidification, acetone extraction, and methanol treatment. The final product was obtained as a white solid (Yield: 43%). A scheme of the synthesis is reported in Figure S1 in the SM.

^1^H NMR (400 MHz, D_2_O): δ 4.08–3.91 (m, 5H), 3.33 (t, 2H), 2.03–1.82 (m, 2H), 1.71–1.47 (m, 4H).

MS (ESI^−^): *m*/*z* 287.1 [M–H]^−^ (C_10_H_16_N_4_O_6_, 288.26 Da).

### 2.2. Synthesis of Copoly DBCO 4%

Copoly DBCO was synthesized via the post-polymerization modification of MCP-2 with 4% NAS, following the procedure reported in [16]. *N*,*N*-dimethylacrylamide, NAS, and 3-(trimethoxysilyl)propyl methacrylate were polymerized in dry THF under argon at 65 °C using AIBN as initiator, as reported in the SM. The polymer was precipitated in petroleum ether and collected.

For DBCO functionalization, Copoly NAS 4% (2 g) was reacted with dibenzocyclooctyne-amine (0.257 g) in dry THF under nitrogen for 5 h. The product was precipitated, filtered, and dried to yield Copoly DBCO 4%. A scheme of the synthesis is shown in Figure S2 in the SM.

### 2.3. Coating of Flat Supports, Silica Coated Beads, and Functionalization with Azido-NTA

Glass and silicon surfaces were activated (ozone or plasma), then coated with 1% w/v polymer (Copoly DBCO or MCP-2) in DI water. MCP-2 coatings included 800 mM ammonium sulfate and were cured at 90 °C.

Silica-coated magnetic beads were similarly treated with polymer solutions, stirred, and washed.

Functionalization was achieved by incubating surfaces or beads with 100 mM azido-NTA in Tris-HCl (pH 8) for 1 h, followed by metal ion loading (Ni^2+^ or Co^2+^, 250 mM). The final surfaces were rinsed and dried. A scheme of the functionalization is reported in the SM (Figure S3).

### 2.4. RPI Sensor

RPI sensor chips were coated with Copoly NTA or MCP-2, as described in Section 2.3. His-tagged α-fetoprotein (AFP), His-tagged SARS-CoV-2 Spike RBD (SP-RBD), and polyclonal anti-α-lactalbumin antibody (pAb-LALBA) were spotted using a non-contact piezoelectric spotter (sciFLEXARRAYER S3, Scienion AG, Berlin, Germany). Proteins were prepared in printing buffer (150 mM Na_2_HPO_4_, pH 8.5, 0.01% w/v sucrose monolaurate) at 0.5 mg/mL for tagged proteins and 1 mg/mL for pAb-LALBA.

After overnight incubation in a humid chamber, MCP-2 chips were blocked (Tris-HCl, pH 8, 10 mM; NaCl 150 mM; ethanolamine 50 mM), rinsed, and dried. Copoly NTA chips were treated with 5 mM imidazole for 5 min, rinsed, and dried. Chips were mounted onto cuvettes and stored at 4 °C.

RPI measurements were performed as in [14], using 1.3 mL PBS 1X as measuring buffer. Spot brightness was analyzed via custom MATLAB software (version R2023B) and converted into surface density (Sd) using the following formula:

(1) $$Sd=\sigma^{*}\sqrt{\frac{u_{s}}{u_{0}}-1}-\delta \sigma$$

where σ*, u_0_, and δσ are known from the physical parameters of the RPI sensor according to [14]. The surface number density of molecules Nd is obtained as Nd = Sd/Mw, where Mw is the mass per molecule.

The average value and standard deviation of the surface density were obtained using at least 15 spots with identical composition. More details on the analysis can be found in the SM.

### 2.5. Binding and Release of Native Antibodies

Protein binding to functionalized magnetic beads was assessed using Rabbit IgG. Beads were coated with Copoly DBCO and functionalized with azido-NTA using either NiSO_4_ or CoSO_4_, as described in Section 2.3. After coating, 100 μL of bead suspension (3.6 mg/mL) was incubated with 100 μL of 500 µg/mL Rabbit IgG in PBS for 1 h at room temperature under stirring (1400 rpm). Binding was evaluated via BCA assay on the collected supernatant. Beads were washed twice with DI water and resuspended in PBS.

Antibody release was tested by incubating beads in 100 µL of either 50 mM EDTA, 250 mM imidazole, or 500 mM imidazole in PBS for 1 h at room temperature. Supernatants were analyzed via UV spectrophotometry (Nanodrop Lite, Thermo Fisher Scientic, Waltham, MA, USA).

To test for reproducibility, three replicates were prepared for each condition.

### 2.6. Binding of His-Tagged Protein

Silica-coated magnetic beads (1 mg/mL) functionalized with Copoly NTA (via NiSO_4_) were washed with PBS and deionized water, then incubated with 250 μL of His-tagged LbHsp90 (300 and 2500 µg/mL in PBS) under stirring for 1 h at room temperature. After incubation, supernatants were analyzed using a BCA assay to quantify unbound protein using a standard calibration curve.

Controls included the following: (i) Copoly NTA without metal, (ii) Copoly DBCO only, both with His-tagged LbHsp90, and (iii) Copoly NTA with metal incubated with BSA (600 µg/mL) to assess non-specific binding. A separate BSA calibration curve was used for quantification. Each condition was tested in triplicate. More details on the experimental setup are reported in the SM.

### 2.7. Immobilization of CRISPR-Cas Enzyme and Oligonucleotide Detection on Beads

The Cas12a–sgRNA1 complex was prepared by incubating 200 nM LbCpf1 with 250 nM sgRNA1 in Cas buffer (20 mM Tris-HCl pH 7.5, 100 mM KCl, 5 mM MgCl_2_, 1 mM DTT, 5% glycerol) at 37 °C for 30 min. The complex was then diluted to 50 nM Cas12a and 62.5 nM sgRNA1 and incubated for 1 h at room temperature with azido-NTA-Ni modified magnetic microbeads (see Section 2.3), in a total volume of 200 μL. After incubation, the beads were washed twice with Cas buffer and exposed to 200 nM DNaseAlert™ substrate for fluorescence detection. Increasing concentrations of target dsDNA (0–200 nM) were added, and fluorescence was measured after 1 min using a DeNovix fluorometer (Cy3 channel), following bead removal. For comparison, the activity of the immobilized complex was evaluated against the free complex in solution, using final concentrations of 10 nM Cas12a and 12.5 nM sgRNA1. All conditions were tested in triplicate.

## 3. Results and Discussion

### 3.1. Design of NTA Copolymer Coating

The accurate orientation of probes immobilized on solid surfaces is critical for ensuring the sensitivity and specificity of biological assays, particularly when working with proteins like antibodies or enzymes, which rely heavily on their three-dimensional structure and dynamic interactions with substrates to function effectively. The flexibility of the MCP-2 polymer formulation introduced by our group allows for the incorporation of a variety of functional groups (such as charged or perfluorinated monomers) to meet specific application needs. Recent advances on this topic have led to the introduction of copolymers which bear click functionalities, allowing for click chemistry reactions for the immobilization of probes [16]. However, although click chemistry offers fast kinetics of reaction and a unique biorthogonality, it still requires probe modification, since the click moieties are not naturally present in probes such as proteins, antibodies, or enzymes.

**Figure 1:**
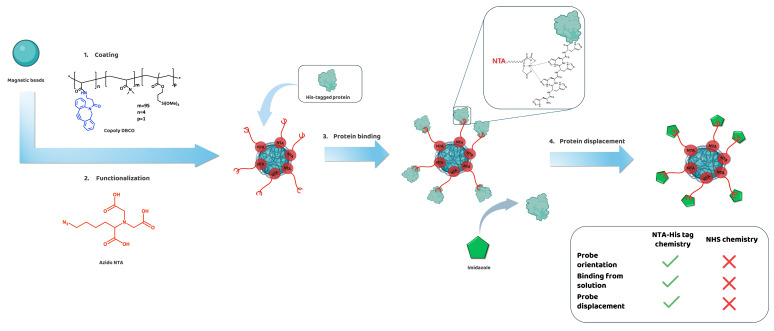
Schematic representation of protein immobilization using Copoly NTA-modified magnetic beads. Magnetic beads are first coated with Copoly DBCO, followed by activation with Azido NTA. The subsequent addition of NiSO_4_ enables coordination with the carboxylic groups of NTA, allowing them to bind the histidine residues of His-tagged proteins. This results in the oriented immobilization of proteins on the bead surface. Finally, the addition of imidazole displaces the bound proteins, effectively releasing them from the magnetic beads.

To this end, we designed and synthetized a new copolymer, which combines the versatility of the MCP click polymers [16] with nitrilotriacetic acid (NTA) chemistry. NTA chemistry leverages the interaction between a divalent ion (such as Ni^2+^ or Co^2+^), coordinated by the three carboxylic acids of NTA, and a short sequence of six histidine residues (His-tag) commonly added to the N- or C-terminus of proteins expressed in bacterial cultures for purification purposes [17]. Since His-tags are an integral part of the protein production process, they are readily available after expression and purification, allowing for their direct use without requiring additional modifications. The placement of the His-tag on the protein ensures a high specificity and oriented probe immobilization in mild conditions (Figure 1) [2,12]. Moreover, the NTA group can also be exploited for the oriented immobilization of antibodies without His-tag, as the Fc region contains a highly conserved area with two histidine residues that are readily recognized by NTA [18].

Initial attempts to introduce commercial amino-NTA onto MCP-2, following a classical post-polymerization modification of the NAS groups, revealed the technical limitations related to the solubilization of amino-NTA, which required a strongly basic pH. This condition inevitably led to the hydrolysis of NAS groups, significantly reducing the yield of the functionalization reaction. To address this issue, the amino group of amino-NTA was converted into an azide through a diazo transfer reaction (see Figure S1 in SM). The resulting azido-NTA molecule required only a mildly basic pH for solubilization, and it was easily inserted into the polymer backbone by replacing the MCP-2 polymer with Copoly DBCO 4% (see Figure S2 in SM) using the SPAAC reaction to graft the azido molecule onto the coated surface. This strategy streamlined the functionalization process, enhancing its speed and reproducibility, resulting in Copoly NTA. The amount of NTA along the polymer chain is limited by the DBCO content in the polymer backbone, which cannot exceed a certain threshold without affecting solubility. Nevertheless, previous studies with the parent polymer MCP-2 (2% functional monomer) demonstrated efficient protein binding (1.2 ng/mm^2^ for IgG) and detection limits (when used in microarray applications) down to the pg/mL and even femtomolar range under optimized conditions [19,20].

Copoly DBCO was previously characterized using FT-IR analysis, which showed the insertion of the DBCO groups after the post-polymerization modification, and using NMR, which highlighted the purity of the obtained product. GPC analysis showed a Mw of 4.83 × 10^3^ g/mol and a polydispersity index of 2.83. Similarly, the morphology and thickness of MCP-2 coatings have been well characterized. The water contact angle was used to highlight the hydrophilicity of the coated surface (40°), while AFM showed a roughness (Rq) of 0.097 nm and a dry thickness of ~2 nm, while Spectral Self-Interference Fluorescence Microscopy SSFM revealed swelling to ~15 nm upon hydration. Dual Polarization Interferometry (DPI) confirmed these values and provided a refractive index of 1.37, mass of 2 ng/mm^2^, and density of 0.2 g/cm^3^. Given the nearly identical composition to the parent MCP-2 polymer—96% dimethylacrylamide, 1% MAPS, and only 3–4% functional monomer (DBCO or NTA)—the new surface is expected to exhibit equivalent physical properties [16,21,22,23].

The coating process is extremely simple and fast, as it merely consists of immersing the support that must be functionalized into a diluted water solution of Copoly DBCO for 30 min following a treatment of 1 h in a water solution of azido-NTA. The activation of the NTA groups is finally achieved by immersing the slides into a solution of NiSO_4_ or CoSO_4_ for 30 min. Overall, the coating process takes up to 2 h, while the literature reports protocols that can take up to 2 days [10]. Our previous studies [24,25] demonstrated the high stability of the coating over time and under various extreme conditions, meaning that the surfaces (both planar or beads) can be prepared in advance and strored. However, the Azido-NTA modification and charge surfaces with metal ions must be performed shortly before use, since the nitrilotriacetic acid group can have a limited stability as it is susceptible to the loss of metal coordination or oxidation over time [5].

The coating was characterized by a label-free optical biosensor and used to functionalize magnetic microbeads (1 μm diameter) with a silica-like surface, which guarantees an optimal coating stability through the formation of covalent bonds with the silanol moieties pending from the polymer backbone.

Several different His-tagged proteins were successfully immobilized on Copoly NTA-coated surfaces, both planar and spherical (magnetic beads). In all cases, similar levels of probe immobilization were achieved, highlighting the robustness, versatility, and efficiency of the His-tag/NTA-mediated binding mechanism.

### 3.2. Immobilization of Proteins on Surface Biosensor

**Figure 2:**
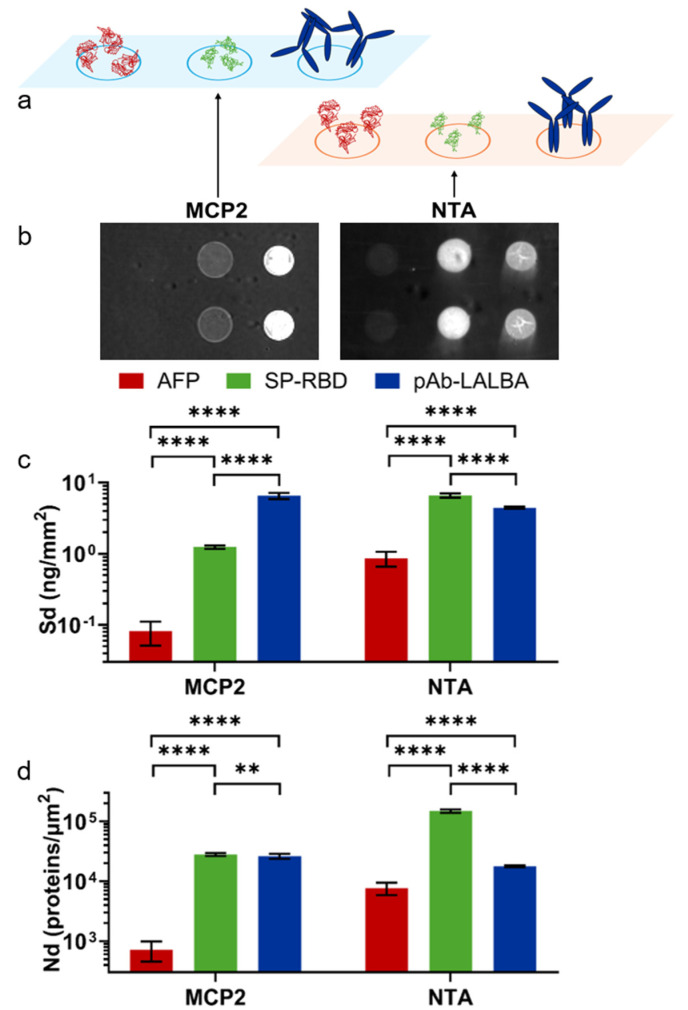
Quantification of immobilization capacity of Copoly NTA. (**a**) Illustration of protein immobilization on MCP-2 and Copoly NTA coatings: random orientation by amine coupling (MCP-2) and oriented immobilization by His-tag (NTA). (**b**) RPI biosensor images of proteins immobilized on the three copolymers: MCP-2 (left image) and NTA (right image). In each image, two spots are displayed for each protein: AFP (left spots), SP-RBD (center spots), and pAb-LALBA (right spots). (**c**) Suface density of proteins on the two copolymer types. (**d**) Number density of proteins corresponding to the data of panel c. In panels c and d, error bars represent standard deviation of at least 15 spots, and asterisks denote statistical significance (** *p* < 0.01; **** *p* < 0.0001) as computed by ANOVA test.

RPI chips were coated with the two different coatings: MCP-2, which provides a random orientation on the surface by amine binding, and Copoly NTA, which provides an oriented immobilization by His-tag (Figure 2a). Three different proteins were immobilized on each chip to evaluate the differences in the immobilization strategies. Two of the selected proteins were His-tagged and provided different isoelectric points (Alpha-Fetoprotein, AFP, pI = 4.5–5.5 and Spike RBD, SP-RBD, pI = 8.9) [26,27]. The other protein is an antibody (anti-α-lactoalbumin, pAb-LALBA), which can bind the NTA group by the native histidine residues despite the lack of a standard His-tag [28]. Figure 2b shows RPI images of the chip after the spotting of the proteins. The spot brightness is converted into the surface density (Sd) of molecules (see Methods), as reported in Figure 2c. On the MCP-2-coated surface, Alpha-Fetoprotein exhibited a very small capture yield, with a Sd = 81 pg/mm^2^, while a signal corresponding to Sd = 1.3 ng/mm^2^ was measured for SARS-CoV-2 Spike RDB, in agreement with the previous work [28]. In contrast, anti-alpha-lactoalbumin antibodies were immobilized with a larger value of Sd. Considering the molecular mass of the three proteins, the measured Sd values on the MCP-2 coating correspond to the number densities Nd reported in Figure 2d, which confirmed the smaller immobilization yield for AFP. This can be ascribed to the acidic behavior of the protein and the limited number of amine groups, which are insufficient for enabling the effective binding to the NAS moieties on the MCP backbone. Furthermore, the spatial distribution of amine residues also plays a critical role. The alkaline SARS-CoV-2 Spike RDB produced a mass surface density on the MCP-2 polymer smaller than the antibody, which, however, results in a similar number density of molecules. Considering the different sizes of SP-RBD and pAb-LALBA, a similar value of Nd suggests that the immobilization yield of SP-RBD may be limited by the accessibility to amine-binding sites on the MCP-2 copolymer. Given the diagnostic significance of these proteins, which hold potential applications in biosensor development, the different immobilization yield of the three proteins, and, in particular, the very low yield of AFP could affect the quantification of the target in solution and lead to false negatives.

A different immobilization behavior is observed on the Copoly NTA-coated surface (Figure 2b,c). The Sd from the His-tagged SARS-CoV-2 Spike RDB increased due to the effective immobilization facilitated by the interaction between the NTA ligand and the exposed histidine tag. An even larger improvement in the Sd of Alpha-Fetoprotein was observed, although the yield remained much smaller than that of the other proteins. In contrast, the number of immobilized antibodies showed a slight decrease relative to the case of MCP-2 coating, which was ascribed to the smaller efficiency of the native histidine residue of the antibody for binding onto NTA relative to the His-tagged proteins. The conversion of Sd to the number density Nd reported in Figure 2d showed an increase of a factor 10 for the AFP immobilization yield and a factor of 5 for SP-RBD. Such a large increase in Nd provides the potential of a corresponding increase in the signal for target binding, and hence increase in sensitivity in diagnostic and biosensor applications. Notably, although the antibody lacks a specific His-tag, the immobilization yield remained similar to that observed for amine binding on the MCP-2 copolymer. To further assess the effect of the oriented immobilization provided by Copoly NTA, we measured, using a label-free RPI biosensor, the binding of α-lactoalbumin to pAb-LALBA immobilized on MCP-2 and Copoly NTA coatings. Analysis of the binding curves revealed very similar equilibrium constants for both coatings, but slightly enhanced kinetic rates for Copoly NTA (Figure S4 in SM). A notable difference between the two coatings was observed in the fraction of active antibodies on the surface. Despite a larger amount of pAb-LALBA being immobilized on MCP-2, the saturation amount of captured α-lactoalbumin was greater on Copoly NTA. Comparing these values showed that the number of pAb-LALBA antibodies on MCP-2 that were active in capturing α-lactoalbumin was approximately half that of Copoly NTA.

Overall, the RPI analysis of the immobilization on the two copolymer coatings demonstrated a much larger efficiency for Copoly NTA with His-tagged proteins. In contrast, for native antibodies lacking a tag, the immobilization efficiency was similar to that of MCP-2. However, the fraction of active antibodies capable of binding their specific antigen was significantly larger on Copoly NTA, a direct consequence of the oriented immobilization.

### 3.3. Capture and Release of Antibody on Magnetic Beads

**Figure 3:**
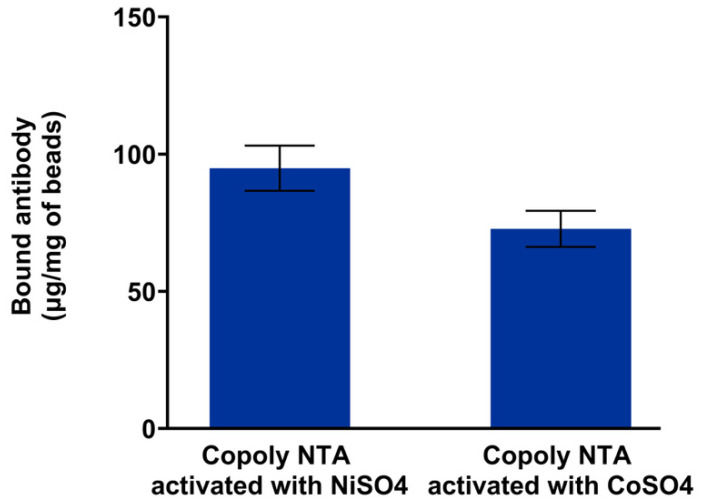
Amount of Rabbit IgG immobilized on magnetic microbeads coated with Copoly NTA activated with both NiSO_4_ or CoSO_4_. The quantity of immobilized IgG is obtained by subtraction of the residual protein concentration remaining in the supernatant after the incubation.

As previously mentioned, the Fc region of antibodies contains two histidine residues that can be utilized for immobilization via metal–NTA chemistry. In this study, magnetic beads (1 µm in diameter) were coated with Copoly DBCO. After functionalization with NTA and the subsequent treatment with NiSO_4_ and CoSO_4_, the beads were incubated with a solution of rabbit IgG. The supernatant was then analyzed using a UV spectrophotometer to measure the residual protein concentration post-incubation, enabling a quantification of the antibody mass bound to the coated and functionalized beads. Data are reported in Figure 3, which also illustrates the differences observed when using two different metal ions, specifically Ni^2+^ and Co^2+^. These metal ions coordinate with histidine residues via the well-defined octahedral complexes formed with NTA, enabling the efficient, reversible, and oriented immobilization of His-tagged proteins. The selection of Ni^2+^ and Co^2+^ is supported by the extensive literature identifying them as the most suitable choices for reliable and functional protein immobilization, offering an optimal balance between binding strength and specificity. In contrast, Cu^2+^ binds too strongly but nonspecifically, while Zn^2+^ forms complexes that are too weak to ensure a stable immobilization [29,30].

The amount of bound antibodies is approximately 90 µg/mg of beads when Ni^2+^ is used as a chelating agent, which facilitates a slightly higher antibody binding capacity compared to Co^2+^ (70 µg/mg of beads). This behavior is well-documented in the literature: in fact, while Ni^2+^ offers a high binding capacity, binding mediated by Co^2+^ offers a greater interaction stability, likely due to the oxidation of the ion to its trivalent form, although it has a slight lower affinity [31]. This is evident from the higher percentage of antibody released during the elution procedure in samples treated with NiSO_4_, indicating a weaker interaction compared to Co^2+^ (see Figure 3). This was further validated by assessing the percentage of antibody eluted following the treatment of the beads with imidazole or EDTA, choosing concentrations commonly used in protocols involving His-tagged protein purification using immobilized metal affinity chromatography (IMAC) [32,33]. Beads treated with Co^2+^ exhibited a lower percentage of antibody release, confirming a more stable binding interaction (see Table 1). In this context, a high concentration of imidazole (500 mM) proved most effective, releasing up to 80% of the bound antibody, whereas EDTA and 250 mM imidazole were only able to release approximately half of the bound antibody.

These results demonstrate that the choice of metal can be adjusted depending on the application and the need for reversible interactions.

### 3.4. Binding of His-Tagged Protein on Magnetic Beads

Magnetic beads are versatile tools widely used for several molecular biology applications, including sample preparation, protein purification, molecular and immunodiagnostics, cell sorting, and biosensing. Furthermore, microbeads are extremely practical to handle, making them suitable also for automatized protocols. Magnetic beads with NTA functionalities are indeed commercially available; however, our approach offers a high flexibility due to the possibility of easily modifying the concentration of NTA groups and the possibility of coating different sizes of beads which might not be available for purchase and which are not suitable for biosensing applications.

**Figure 4:**
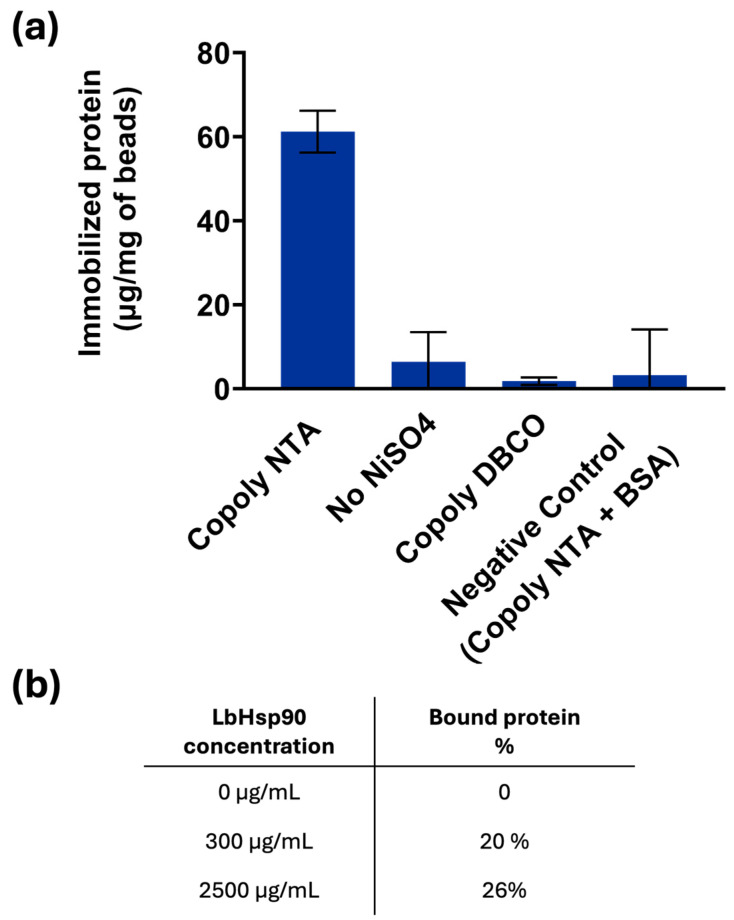
(**a**) Amount of LbHsp90 protein immobilized on the functionalized magnetic microbeads coated with Copoly NTA. BSA was used to evaluate non-specific binding; (**b**) percentage of LbHsp90 bound to magnetic microbeads coated with Copoly NTA, relative to free LbHsp90 in solution.

To prove the efficacy of the immobilization of probes with NTA-Ni chemistry and the versatility in immobilizing several different His-tagged proteins, protein binding assays were conducted employing as a model the His-tagged recombinant Heat Shock Protein 90 (Hsp90) alpha protein. In particular, the Hsp90 from *Leishmania braziliensis* (LbHsp90) was cloned and expressed as a recombinant protein in *Escherichia coli* with six histidine residues at the *N*-terminal domain. After magnetic bead coating and functionalization, they were incubated with solutions of His-tagged LbHsp90 (300 and 2500 μg/mL). A BCA assay (a common colorimetric method for protein quantification) was employed to quantify the protein concentration in solution before and after the incubation. The amount of protein captured by the NTA-Ni modified surface was calculated by subtracting the concentration of the protein remaining in the supernatant after the incubation from the initial protein concentration; data are reported in Figure 4.

Based on the previous results, only NiSO_4_ was used as a chelating agent, and, as illustrated in the graph of Figure 4, its presence is mandatory to bind His-tagged LbHsp90 to the beads coated with Copoly NTA. In particular, it is observed that the amount of protein bound to the beads is 60 μg/mg of beads, consistent with the amount of protein bound to the Copoly NTA-coated surfaces in the previous paragraphs. Although the theoretical binding capacity of the beads was estimated to be approximately 400 µg of LbHsp90 per mg of beads, the experimental data suggest that the actual amount of protein bound is significantly lower. This discrepancy is likely due to the steric hindrance caused by the size and conformation of the LbHsp90 protein. In fact, based on calculations of the projected surface area of the protein, it is estimated that only a few tens of micrograms per mg of beads can realistically be accommodated on the bead surface.

The experimentally observed binding of 60 µg/mg beads supports this interpretation, indicating that steric constraints limit the accessibility of functional groups. Nevertheless, the presence of a defined orientation, potentially facilitated by linker-mediated attachments (six histidine residues), appears to enhance binding efficiency slightly. Even though the linker is short, it may provide sufficient spatial separation to reduce steric clashes and allow more proteins to bind than would be possible with a random orientation alone.

This hypothesis is further supported by an experiment conducted using a high concentration of LbHsp90 (2.5 mg/mL), which is in large excess relative to the number of functional groups theoretically available for binding. Under these conditions, 26% of the initial protein was bound, compared to 20% when using 300 µg/mL of protein. This result suggests that the system approaches saturation at higher protein concentrations, reinforcing the idea that steric hindrance, rather than functional group availability, is the primary limiting factor in protein binding.

The absence of azido-NTA resulted in a complete lack of LbHspP90 binding: in fact, Copoly DBCO and the native protein do not have any compatible functionalities to react with each other. Similarly, the single presence of the azido-NTA molecule is not sufficient to bind the histidine of the LbHsp90, since the binding is mediated by the bivalent ion as Ni^2+^, as depicted in Figure 4. Negligible binding was observed with a protein lacking histidine residues (such as BSA), highlighting not only the specificity of the NTA-Ni-protein interaction, but also the anti-fouling properties of the coating polymer, which effectively prevents non-specific binding. The anti-fouling properties of the parent polymer MCP-2—expected to be equivalent to those of the coating presented here due to their similar composition—have been previously evaluated and widely exploited. These include applications in highly sensitive microarray assays (both fluorescence- and interference-based) [34,35,36], as well as in improved protein separation by capillary electrophoresis, owing to the minimal interaction between proteins and the coated fused silica capillary walls [37]. This unique feature of the polymer-coated beads is crucial for the development of biosensing systems, as minimizing non-specific binding significantly enhances the sensitivity of the assay.

### 3.5. Immobilization of CRISPR-Cas Enzyme and Oligonucleotide Detection on Beads

While protein immobilization onto a surface is inherently challenging, enzyme immobilization is particularly difficult. Enzymes depend not only on their precise three-dimensional structure but also on dynamic interactions with substrates. Constraining enzymes onto a solid support can disrupt these interactions and compromise their catalytic efficiency. To demonstrate the efficacy of this coating in maintaining the structure and the activity of proteins, we tested the immobilization of the CRISPR–Cas (clustered regularly interspaced short palindromic repeats–Cas associated) enzyme system, whose His-tagged form is commercially available (His-tag Cas12a). The CRISPR–Cas system is an adaptive immune mechanism in prokaryotes that protects against phage infections by storing viral DNA in bacterial host chromosomes as a form of memory [38]. In recent years, CRISPR-Cas-based diagnostic tools are emerging for the detection of infectious diseases because of their high specificity and high sensitivity [39,40,41]. Specifically, the CRISPR-Cas 12a isoform possesses peculiar features which make it suitable for diagnostic applications. In fact, this enzyme works by forming a complex with an RNA sequence (sgRNA) that recognizes a complementary sequence of dsDNA, and, upon recognition, this isoform acquires the ability to cut non-specific sequences of ssDNA. This feature has been exploited to generate a fluorescent signal by adding a short sequence of ssDNA bearing both a fluorophore and a quencher at the two ends. Upon DNA recognition and interaction with the CRISPR-Cas, this short sequence is excised: the fluorophore is separated from the quencher and a signal is generated.

**Figure 5:**
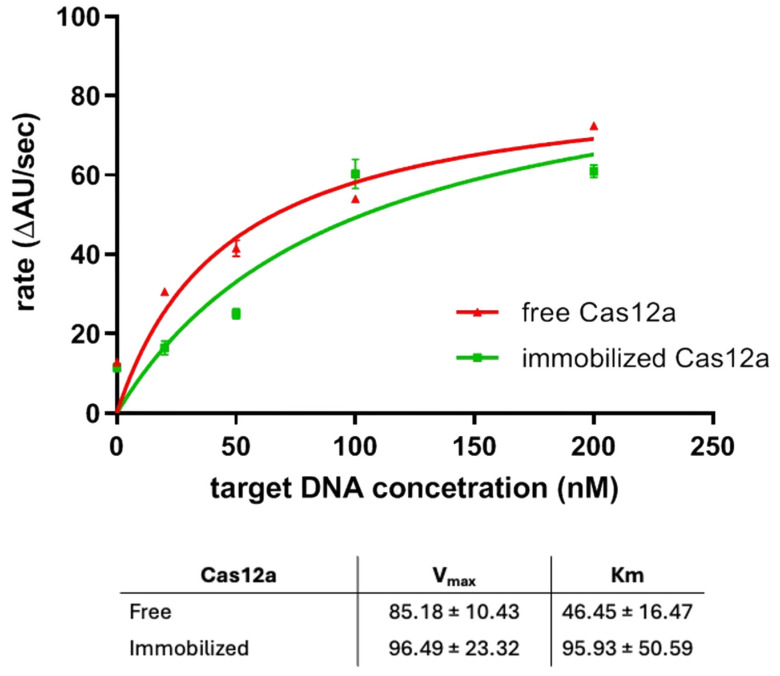
Michaelis–Menten kinetics of CRISPR-Cas12a in free solution (red line; triangles) and immobilized on bead surfaces (green line; squares). The reaction rate (ΔAU/sec), derived from the fluorescence intensity of a cleaved ssDNA reporter, was measured after 1 min of incubation with increasing concentrations of target dsDNA (0–200 nM).

The Cas12a-sgRNA complex was immobilized on magnetic microbeads coated with Copoly NTA activated with NiSO_4_ as reported in Section 2.3. In this case, Ni^2+^ was chosen to avoid too strong of an interaction with the surface, which might decrease the enzyme activity. Briefly, after incubating the Cas12a with the sgRNA for 30 min in Cas buffer, the complex was used to resuspend coated and activated magnetic microbeads; after rinsing, the complementary target dsDNA was added to the microbead suspension together with the fluorescence reporter ssDNA sequence. Increasing concentrations of the target dsDNA (ranging from 0 to 200 µM) were added, and the fluorescence was measured after 1 min of reaction. The activity of CRISPR-Cas12a immobilized on the bead surface was compared to that of the enzyme in solution. To ensure a fair comparison, the concentration of free CRISPR-Cas12a was reduced from the standard 50 nM (as recommended by the supplier) to 10 nM, reflecting the estimated amount of enzyme immobilized on the beads. In fact, previous experiments have shown that the immobilized fraction corresponds to approximately 20% of the enzyme present in solution. This adjustment allowed for a more accurate evaluation of the relative enzymatic activities under comparable conditions. The collected signals were fitted using the Michaelis–Menten kinetic model and are presented in Figure 5.

The data show that the enzyme immobilized on the beads via the NTA ligand conserves its activity (Figure 5, green line) when compared to the activity of the free CRISPR-Cas12a in (Figure 5, red line). Both curves show the same trend, with the typical saturation reached in both cases at the same substrate concentration. The V_max_ values are very similar, indicating that the maximum catalytic activity of CRISPR-Cas12a is well preserved upon immobilization; however, the K_m_ for the immobilized enzyme is approximately double that of the free enzyme, suggesting a lower apparent affinity for the substrate. This could be due to diffusion limitations, steric hindrance, or microenvironmental effects introduced by the immobilization matrix. Overall, the data strongly support that CRISPR-Cas12a retains its catalytic efficiency upon immobilization. This validates the effectiveness of the immobilization strategy on Copoly NTA-coated magnetic beads and confirms that the enzyme remains functionally active and properly oriented on the bead surface.

It is also important to note that the suggested strategy enables the immobilization of bioprobes directly from a solution, an approach that is not easily achievable with classical amine–NHS ester chemistry due to the rapid hydrolysis of NHS esters in aqueous environments. Our previous studies [16,42] have already highlighted the need for different immobilization strategies in cases where probes must be coupled directly from a solution, without intermediate drying or concentration steps, introducing click chemistry as an alternative. In this context the NTA–His interaction, despite all constraints, proved equally effective in promoting immobilization and preserving enzyme functionality.

## 4. Conclusion

In this study, we developed and optimized a novel protein immobilization strategy that combines the versatility of MCP click polymers with NTA-based affinity chemistry. The results demonstrated that this approach ensures the highly specific, oriented, and reversible immobilization of different His-tagged proteins under mild conditions, while preserving their biological activity. The introduction of azido-NTA chemistry significantly improved the functionalization efficiency by addressing the solubility issues encountered with amino-NTA, leading to a more reproducible and streamlined process. Different metal ions commonly recommended in the literature—such as Ni^2+^ and Co^2+^—were evaluated for their ability to chelate NTA groups. Their selection was shown to significantly influence both binding efficiency and reversibility, enabling tunable interactions that can be optimized for different applications.

The comparative RPI analysis demonstrated that Copoly NTA coating significantly enhances the immobilization efficiency of His-tagged proteins, while maintaining a comparable performance to MCP-2 for native antibodies, highlighting its superior potential for sensitive biosensor applications.

We validated this approach by functionalizing magnetic beads and successfully immobilizing His-tagged proteins like LbHsp90 and enzymes such as CRISPR-Cas12a. The activity data obtained for immobilized CRISPR-Cas12a demonstrate that the enzyme retains its full functionality upon binding. The kinetic parameters—Vmax and Km—are remarkably similar to those of the free enzyme in solution, strongly supporting the effectiveness of the immobilization strategy and the correct orientation of the protein mediated by the Copoly NTA coating.

## Figures and Tables

**Table 1:** Percentage of Rabbit IgG released from Copoly NTA activated with both NiSO_4_ or CoSO_4_. Three different releasing solutions, known to chelate the metal ion (EDTA) or to compete with the histidine residue interaction, were tested.

	Releasing Solution		
	EDTA 50 mM	Imidazole 250 mM	Imidazole 500 mM
Azido-NTA with NiSO_4_	57.6%	55.8%	82.0%
Azido -NTA with CoSO_4_	46.0%	39.0%	62.2%

## Data Availability

Data is contained within the article.

## Supplementary Materials

The following supporting information can be downloaded at: https://www.mdpi.com/article/10.3390/bios15100670/s1, Figure S1: Schematic synthesis of azido-NTA; Figure S2: Schematic synthesis of MCP-2 polymer and the relative post-polymerization modification; Figure S3: Scheme of the surface coating with Copoly NTA; Figure S4: Binding parameters measured by RPI biosensor.

## Abbreviations

The following abbreviations are used in this manuscript:

NTA	Tetradentate nitrilotriacetic acid
pAb-LALBA	Anti-α-lactoalbumin
AFP	Alpha-Fetoprotein
DBCO	Dibenzocyclooctyne
RPI	Reflective Phantom Interface
DMA	*N*, *N*-dimethylacrylamide
NAS	*N*-acryloyloxysuccinimide
MAPS	3-(Trimethoxysilyl)propyl methacrylate)
