# Supplementary material for: Affinity-Based Copolymer Coating for Oriented Protein Immobilization in Biosensor Development
Source: Biosensors (Basel). 2025 Oct 4;15(10):670. doi: 10.3390/bios15100670 (PMC12564766; doi:10.3390/bios15100670)
Supplement: Supplementary file 1 [file biosensors-15-00670-s001.zip › biosensors-3754540-supplementary.pdf]

Supporting information

Affinity-based copolymer coating for oriented protein immobilization in biosensor development

Synthesis of (S)-2,2′-((5-azido-1-carboxypentyl)azanediyl)diacetic acid

In a 50 mL two-neck round bottomed flask, (*N-N*-bis[carbossimetil]) L-lysine (0.581 g, 2.21 mmol) was dissolved in 17,5 mL of DI water to a final concentration of 130 mM. Then, $CuSO_4$ (0.012 g, 0.044 mmol) and K_2CO_3 (1.38 g, 9.965 mmol) were added in sequence and stirred until complete dissolution. Subsequently, 1H-imidazole-1-sulfonyl azide (0.557 g, 2.66 mmol) and acetonitrile (5 mL) were added to the reaction. The resulting mixture was stirred overnight at room temperature. The solvent was removed by evaporation under reduced pressure, and the resulting crude was dissolved in water (7.5 mL) and then washed with 3 mL of acetonitrile three times. The aqueous layer was acidified with chloridric acid to pH=2 and then extracted twice with acetone (5 mL). Finally, the organic phase was then concentrated under reduced pressure. The crude was then dissolved with methanol (3mL) and, after removal of the precipitate through filtration, concentrated under reduced pressure. The yellowish solid obtained was then washed two times with water (1 mL) and finally dried in a vacuum oven overnight at room temperature, obtaining the desired product as a white solid (see Figure 1). Yield 43%. 1H NMR (400 MHz, D_2O) δ 4.08 – 3.91 (m, 5H), 3.33 (t, J = 6.5 Hz, 2 H), 2.03 – 1.82 (m, 2H), 1.71 – 1.47 (m, 4H). *The compound exhibited a peak at m/z 287.1 corresponding to the deprotonated molecular ion [M–H]⁻, consistent with the calculated mass of* $C_{10}H_{16}N_4O_6$ *(288.26 Da). A scheme of the synthesis is reported in Figure S1.*

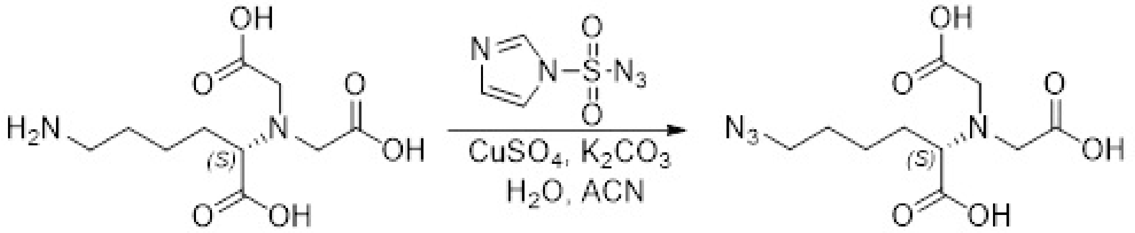

Figure 1. Schematic synthesis of azido-NTA.

2.2. Synthesis of Copoly DBCO 4%

Copoly DBCO was synthesized following the procedure reported in [16], starting by post-polymerization modification of the MCP-2 polymer. However, in this case, the molar fraction of the NAS monomer was increased to 4%. Briefly, N,N-dimethylacrylamide (1.978 g, 19.95 mmol), N-acryloyloxysuccinimide (0.142 g, 0.84 mmol), and 3-(Trimethoxysilyl)propyl methacrylate) (0.052 g, 0.21 mmol) were dissolved in 10 mL of dry tetrahydrofuran (THF) after degassing with argon. To initiate the polymerization, α, α′-Azoisobutyronitrile (5 mg) was added to the reaction mixture, which was heated to 65°C for 2

hours. The crude was diluted 1:1 with dry THF, precipitated in petroleum ether (200 mL) while stirring, and finally collected by filtration as a white powder.

Post-polymerization modification of the NAS groups into DBCO was performed by dissolving the collected Copoly NAS 4% (2 g, 0.19 mmol) in 20 mL of dry THF. Under a nitrogen stream, dibenzocyclooctyne-amine (0.257 g, 0,929 mmol) was added to the solution, and the resulting mixture was left stirring for 5 hours at room temperature. The crude was precipitated in petroleum ether (200 mL) while stirring, filtered over a Buckner funnel to obtain a white powder, and dried under a vacuum to remove all the solvent. A scheme of the synthesis is shown in Figure S 2.

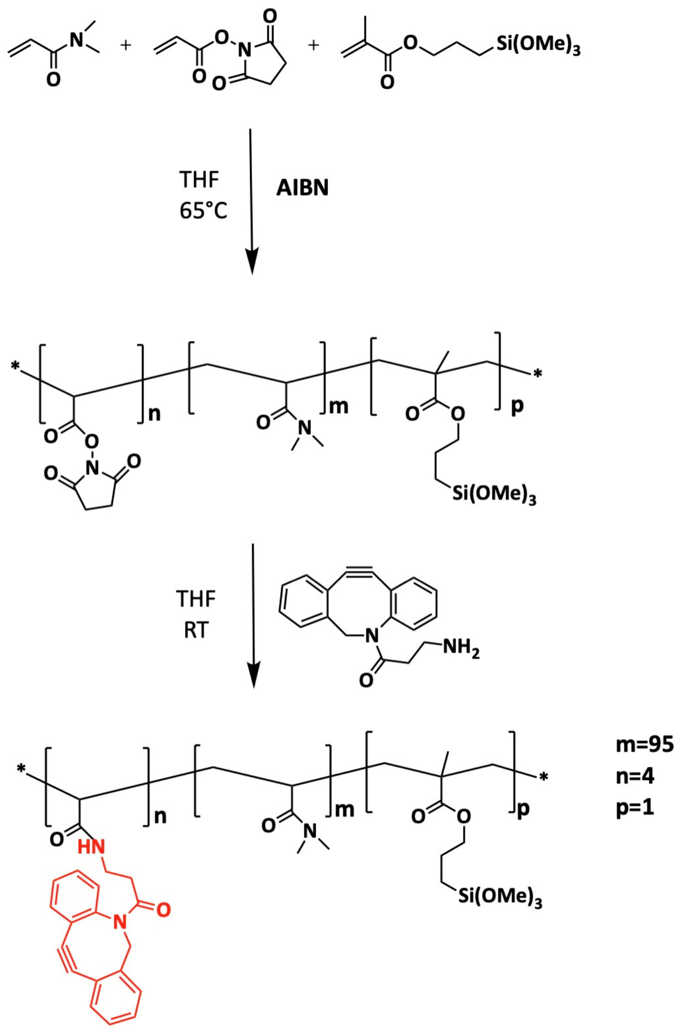

Figure 2. Schematic synthesis of MCP-2 polymer and the relative post-polymerization modification with dibenzocyclooctyne-amine to Copoly DBCO 4%.

2.3. Coating of flat supports, silica coated beads, and functionalization with azido-NTA

RPI glass prisms with SiO2 anti-reflective coating and silicon slides (with a 100 nm thermally grown silicon oxide layer) were activated using either ozone or 15 minutes oxygen plasma treatment. The surfaces were then immersed for 30 minutes at room temperature in a 1% w/v solution of the designated polymer dissolved in DI water (Copoly DBCO or MCP-2). In the case of the MCP-2 copolymer, ammonium sulphate was added to the coating solution to a final concentration of 800 mM. The supports were rinsed with DI water and dried using a nitrogen stream. For MCP-2, an additional curing step was applied by heating the coated surface at 90°C for 15 minutes to stabilize the coating.

A similar procedure was used for the coating of magnetic beads. In particular, silica-coated beads (40 mg/mL) were carefully resuspended by vortexing for 30 seconds; then, 45 µl of the suspension was collected and the supernatant was discarded with the help of a magnet stand. The beads were rinsed twice using 200 µL of DI water and then resuspended with a 1% w/v DI water solution of the designated polymer, or in 800mM ammonium sulphate solution containing 1% w/v of MCP-2 polymer. The bead suspension was stirred at 1400 rpm for 30 minutes, then was rinsed twice with DI water using a magnet stand to eliminate the supernatant.

Functionalization with the azido NTA was performed by immersing the slides into a 100 mM azido-NTA solution in Tris-HCl buffer 1M pH 8 for 1 hour at room temperature. The surface was rinsed with DI water, dried with a nitrogen stream, and then incubated with 250 mM solutions of NiSO4 or CoSO4 in DI water for 30 minutes at room temperature. The slides were rinsed with DI water and dried with a nitrogen stream. Similarly, the supernatant of 45 µL coated magnetic bead suspension (40 mg/ml) was discarded, and the beads were washed twice with 200 ul of DI water, then resuspended with 500 µL of azido-NTA in Tris-HCl buffer 1M pH 8 and stirred at 1200 rpm for 1 hour at room temperature; then, they were rinsed twice with DI water using a magnetic stand to remove the supernatant. A scheme of this process is reported in Figure S 3.

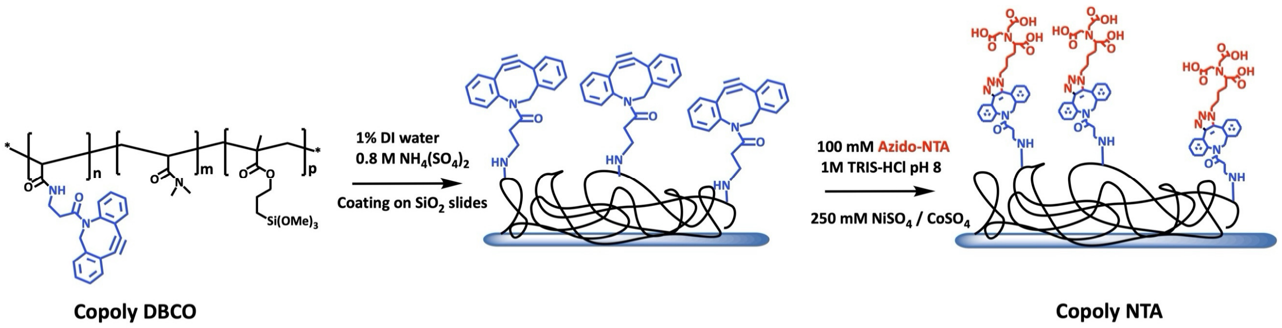

Figure 3. Scheme of the surface coating with Copoly NTA. Firstly, surfaces are coated with Copoly DBCO, then surfaces are treated with azido-NTA in presence of NiSO4 or CoSO4. A click chemistry reaction (SPAAC) occurs between the DBCO groups on the surface and the azide group of the NTA; therefore, a new functionality is introduced onto the surface.

2.4. RPI Sensor

The RPI surface was coated with Copoly NTA and MCP-2 as reported in Section 2.3. Both native and His-tagged proteins were deposited on the surface of the RPI sensor by means of a non-contact piezoelectric spotter (sciFLEXARRAYER S3; Scienion AG, Berlin, Germany). In particular, His-tagged α-feto protein (AFP) and His-tagged SARS-CoV-2 Spike protein RDB (SP-RBD) were dissolved in printing buffer (150 mM Na_2HPO_4, pH 8.5, and 0.01% w/v sucrose monolaurate) at a final concentration of 0.5 mg/mL, while the polyclonal antibody against α-lactalbumin (pAb-LALBA) was dissolved in printing buffer at a final concentration of 1 mg/mL. After overnight incubation in a humid chamber, the MCP-2-coated chip was immersed for 1 hour at room temperature in blocking buffer (Tris-HCl, pH 8, 10 mM, NaCl 150 mM, ethanolamine 50 mM), then rinsed with distilled water and finally dried with a nitrogen stream. On the contrary, the chips functionalized with Copoly NTA, were immersed in a 5 mM solution of imidazole for 5 minutes at room temperature, then rinsed with Di water and dried with nitrogen stream. The sensor cartridges were prepared by gluing the glass chips onto the inner wall of 1 cm plastic cuvettes. The cartridges were stored at 4 ◦C before use.

The RPI measurements were performed using the apparatus and the analysis algorithm described in [17]. The sensor cartridges were filled with 1.3 mL of measuring buffer (PBS 1X). The RPI images of the spotted surface were analyzed by a custom MATLAB program (The MathWorks, Natick, MA, USA) to obtain the brightness of each spot and converted into the total mass surface density of molecules.

The conversion of the brightness of the RPI image pixels into surface density (Sd) was performed according to the following equation:

$$Sd = \sigma^* \sqrt{\frac{u_S}{u_0} - 1} - \delta\sigma \quad (1)$$

where σ^*, u_0, and $\delta\sigma$ are known from the physical parameters of the RPI sensor according to [17]. The surface number density of molecules Nd is obtained as Nd = Sd/Mw, where Mw is the mass per molecule.

The average value and standard deviation of the surface density was obtained by at least 15 spots with identical composition.

2.5. Binding and release of native antibodies

The binding of proteins to the surface of functionalized magnetic beads was performed using a Rabbit IgG antibody. Copoly DBCO was tested as a coating for the beads following the procedure reported in Section 2.3; functionalization with azido-NTA was performed using both $NiSO_4$ and Co SO_4. After coating and activation, 100 µL of the bead suspension (3.6 mg/mL) was collected, and the supernatant was discarded using a magnetic stand. Then, they were resuspended in 100 µL of a 500 µg/mL Rabbit IgG solution in PBS and stirred at 1400 rpm for 1 hour at room temperature. The supernatant was collected and analyzed using a BCA assay to evaluate the binding of the Rabbit IgG to the functionalized beads. Briefly, 25 µL of sample was added to 200 µL of working reagent (WR) solution (prepared according to Pierce™

BCA Protein Assay Kit protocol). The beads were finally rinsed twice with 200 μL of DI water and suspended in 100 μL of PBS.

Release of the bound antibody was performed by resuspending the beads in 100 μL of three different solutions: i) 50 mM EDTA in PBS, ii) 250 mM imidazole, and iii) 500 mM imidazole. The three suspensions were stirred at 1400 rpm for 1 hour at room temperature. The supernatant was collected and analyzed with a UV spectrophotometer (Nanodrop). To test for reproducibility, three replicates were prepared for each condition.

2.6. Binding of His-Tagged protein

A silica-coated magnetic microbead suspension (1 mg/mL) coated in Copoly NTA (using $NiSO_4$) was washed twice with 200 μL of PBS. The beads were incubated with 250 μL of a 300 ug/mL solution in PBS of His-tagged HSP90 and stirred for 1 hour at room temperature. The beads were then re-collected, and the supernatant was analyzed with a BCA assay to evaluate the remaining protein in solution. Briefly, 25 μL of sample was added to a microtiter plate with 200 μL of working reagent (WR) solution (prepared according to Pierce™ BCA Protein Assay Kit protocol). The plate was then incubated at 37°C for 30 min and then read at 562 nm wavelength with a Bio-Rad plate reader. The data on absorbance obtained in the assay were converted to protein concentrations through a calibration curve determined with solutions at known concentrations of protein. Different controls were included in the experiment: i) beads coated with Copoly NTA without using any metal, and incubated with His-tagged HSP90; ii) beads only coated with Copoly DBCO and incubated with His-tagged HSP90; and iii) beads coated with Copoly NTA with metal and incubated with 600 ug/mL of Bovine serum albumin in PBS (BSA, not presenting the His-tag sequence), to evaluate non-specific binding.

To quantify the amounts of BSA, another calibration curve with different BSA concentrations (0.05, 0.1, 0.5 and 1 mg/mL) was prepared. To test for reproducibility, three replicates were prepared for each condition.

2.7. Immobilization of CRISPR-Cas enzyme and oligonucleotide detection on beads

The Cas12a-sgrNA complex was prepared by incubating in a vial 200 nM LbCpf1 with 250 nM sgRNA1 (previously dissolved in RNA-Free water) at 37°C for 30 minutes while stirring in Cas buffer (20 mM Tris-HCl, pH 7.5, 100 mM KCl, 5 mM $MgCl_2$, 1 mM DTT, and 5% glycerol). The Cas and the sgRNA were diluted respectively to 50 nM and 62.5 nM in Cas buffer and incubated for 1h at room temperature at 1000 rpm, mixing with azido-NTA-Ni modified magnetic microbeads (see Section 2.3) in a total volume of 200 μL. The solution was then removed, and the beads were re-collected and washed with Cas buffer (2x200 μL). The beads were subsequently incubated with a solution of 100 nM dsDNA target and 200 nM of DNaseAlert substrate™. Fluorescence intensity was measured with a Denovix fluorometer in the channel of Cy3, immediately after the addition of the solution

containing the dsDNA target, collecting before the bead's precipitation on the bottom of the vial. The solution was then incubated for 1.5 h at 37 °C at 1000 rpm mixing, measuring the fluorescence at 30 min time intervals. A vial containing the beads with the Cas12a-sgRNA complex immobilized and 200 nM solution DNaseAlert substrate™ without dsDNA target was used as a negative control. The activity of the Cas12a-sgRNA complex on microbeads was compared to the enzyme in solution. For each condition, three replicates were prepared.

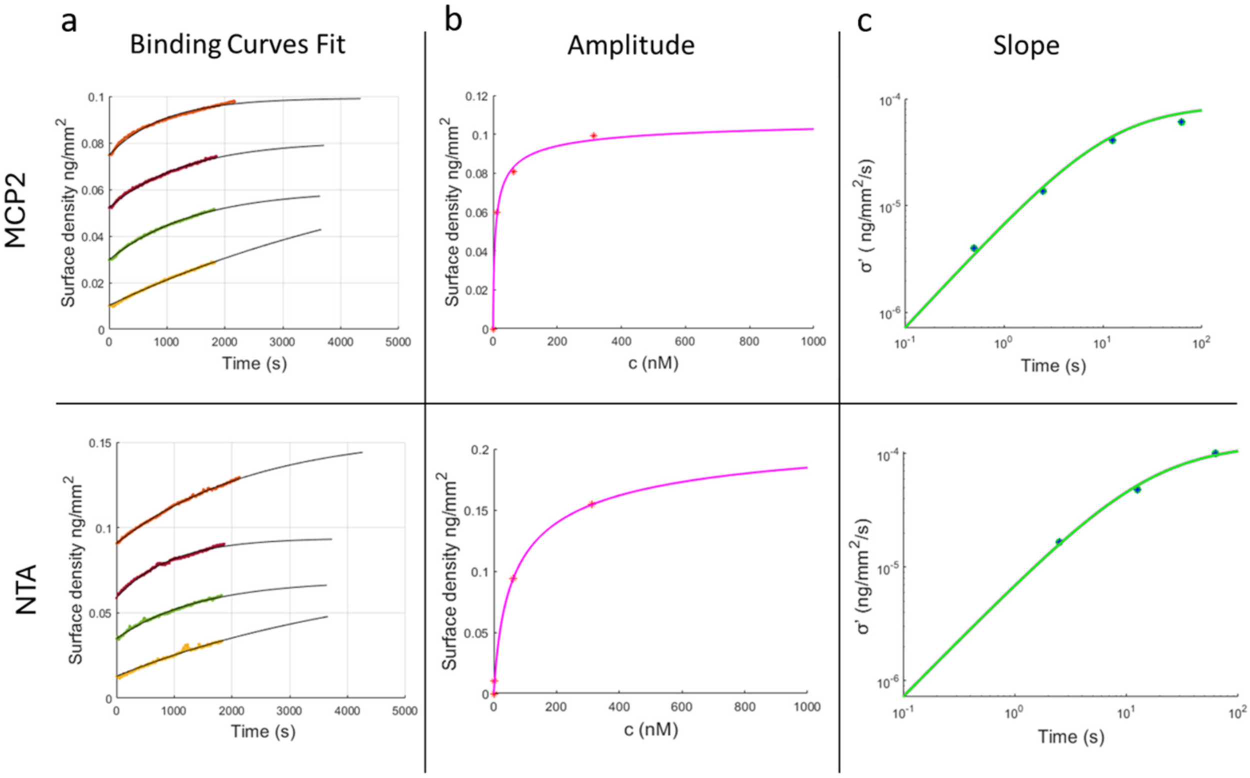

d

Coating	K_d (nM)	k_{off} (s^{-1})	k_{on} (s^{-1} nM^{-1})	Sd_i (ng mm^{-2})	Sd_f (ng mm^{-2})	Sd_n (ng mm^{-2})
**MCP2**	11.5 (±2.2)	$1.4\cdot10^{-3}$ ($\pm3\cdot10^{-4}$)	$1.2\cdot10^{-4}$ ($\pm2\cdot10^{-5}$)	6.5 (±0.6)	0.097 (±0.02)	1
**NTA**	13.8 (±2.8)	$3.7\cdot10^{-3}$ ($\pm8\cdot10^{-4}$)	$2.7\cdot10^{-4}$ ($\pm6\cdot10^{-5}$)	4.4 (±0.16)	0.13 (±0.008)	1.95

Figure 4. Binding parameters measured by RPI biosensor. (a) RPI binding curves measured on MCP2 (top) or NTA polymer (bottom) on pAb-LALBA spots for increasing concentrations of α-lactoalbumin in solution: 2.5 nM (yellow), 12.5 nM (green), 62.5 nM (red), 312.5 nM (orange). The black curves are fits to the data by single exponential growth functions. (b) Equilibrium asymptotic amplitudes obtained from exponential fits to the binding curves in panel (a). Lines represent fits with Langmuir isotherms corrected for a small repulsion at large surface coverage as described in [https://doi.org/10.1093/nar/gkae576]. (c) Initial slopes of the binding curves obtained from the exponential fit shown in (a). Lines represent linear fits corrected for a progressive reduction of the binding kinetics at large surface coverage as described in [https://doi.org/10.1093/nar/gkae576]. (d) Summary table of equilibrium and kinetic parameters obtained from the fit reported in

panels b and c. The table also reports the initial surface density of immobilized pAb-LALBA (Sdi), saturation surface density of α-lactoalbumin binding on pAb-LALBA (Sfi), and corresponding normalized surface density of α-lactoalbumin (Sdn) relative to the value of Sfi for MCP2. The reported uncertainties represent the standard deviations of the parameters computed on at least three different groups of spots of the same type.
